# Maternal depression in Syrian refugee women recently moved to Canada: a preliminary study

**DOI:** 10.1186/s12884-017-1433-2

**Published:** 2017-07-24

**Authors:** Asma Ahmed, Angela Bowen, Cindy Xin Feng

**Affiliations:** 1 School of Public Health, University of Saskatchewan, Saskatoon, Canada; 2 College of Nursing, University of Saskatchewan, 104 Clinic Place, Health Sciences Building, Room 4246, Saskatoon, SK S7N 2Z4 Canada; 3 School of Public Health, University of Saskatchewan, 104 Clinic Place, Health Sciences Building, Room 3338, Saskatoon, SK S7N 2Z4 Canada

**Keywords:** Syrian refugee, Refugee women, Mental health, Pregnancy, Postpartum health, Maternal depression, Healthcare services access

## Abstract

**Background:**

Refugee women are almost five times more likely to develop postpartum depression than Canadian-born women. This can be attributed to various difficulties they faced before coming to Canada as well as during resettlement. Moreover, refugee women usually face many obstacles when accessing health services, including language and cultural barriers, as well as unique help-seeking behaviors that are influenced by various cultural and practical factors. There has been a recent, rapid influx of Syrian refugees to Canada, and many of them are childbearing women. However, little is known about the experiences that these women have encountered pre- and post-resettlement, and their perceptions of mental health issues. Thus, there is an urgent need to understand refugee women’s experiences of having a baby in Canada from a mental health perspective.

**Methods:**

A mixed methods research design included 12 Syrian refugee women who migrated to Saskatoon in 2015–16 and who were either pregnant or 1 year postpartum. The data were collected during a single focus group discussion and a structured questionnaire.

**Results:**

Our results showed that more than half of participants have depressive symptoms, half of them have anxiety symptoms, and one sixth have PTSD symptoms. Three major themes emerged from the qualitative data: 1) Understanding of maternal depression; 2) Protective factors for mental health; and 3) Barriers to mental health services.

**Conclusions:**

Maternal depression is an important feature in Syrian refugee women recently resettled in Canada. Reuniting these women with their families and engaging them in culturally appropriate support programs may improve their mental health outcomes.

**Electronic supplementary material:**

The online version of this article (doi:10.1186/s12884-017-1433-2) contains supplementary material, which is available to authorized users.

## Background

For most women, having a baby is a joyful experience; but, for 10–19% of pregnant women and 9–14% of women postnatally worldwide, it can bring stress, anxiety, depression, and moodiness [1]. These figures are more pronounced in developing nations, where between 15 and 57% of women experience maternal depression (depression during pregnancy and/or up to one-year postpartum) [2]. In Canada, refugee, immigrant, and asylum-seeking women are reported to experience almost five times more postpartum depression than Canadian-born women [3]. Maternal depression is associated with poor outcomes for the mother, her partner relationship, mother-infant bonding, and child development, as well as huge societal and economic burden [4, 5].

Most studies that have examined maternal depression among immigrant women did not distinguish between immigrants, refugees, and asylum seekers [6–8]; however, it is important to make that distinction due to the different circumstances the women face in their relocation and settlement. Immigrant people often choose to migrate to another country due to educational, economic, or family reasons, with the hope to achieve a better life for themselves and their families. Refugees on the other hand, are usually forced to leave their country because of fear for their safety. Asylum-seekers are fleeing a threatening situation in their home country and usually seek protection after their arrival in the new country [9].

Refugees may live in refugee camps and experience various forms of violence (including sexual and physical violence and torture), disruption of family relationships and/or death of loved ones, combined with feelings of uncertainty about the future [8, 10]. Refugees may continue to experience difficulties related to their past trauma as they are resettled, including worries about family and friends still in war zones or refugee camps [11–13], and unfamiliarity with the systems and regulations in their new country [10]. Resettlement may be associated with loss of identity and social status [14]. Refugees are often unemployed or have low paying jobs compared to what they had in their home countries and may face discrimination in the workplace, which can cause stress for the whole family [6].

The usual roles of refugee women change in their new country; consequently, they can feel powerless, and as they get separated from their social support network, they become increasingly dependent on their male partner, which make them more vulnerable to abuse [15]. Language difficulties can worsen social isolation and act as a barrier to engagement and adaptation in their new country and limit their participation in social events [16]. In some cultures, women receive strong support particularly from female relatives during the postpartum period, but the loss of these supports, traditional customs, and cultural practices related to childbirth may leave refugee women feeling socially isolated and more susceptible to develop depression [3, 6, 17]. All of these factors may contribute to poor mental health [18, 19].

Some cultures, such as some Asian cultures, do not view depression as a medical problem, but rather a natural consequence of childbirth, and thus women with these cultural backgrounds were unlikely to seek help [20]. Lack of information or resources about maternal depression can also prevent women from seeking help [21, 22]. The stigma associated with mental health problems may also be a barrier to seeking help [22, 23]. Many refugee women hide their symptoms from family and friends and from health care providers for fears of stigmatization, fear that their symptoms will interfere with their family’s harmony, fear of disclosure, fear of community judgment, or from social pressure [6].

Most Syrian refugees are of Muslim faith. For many Muslims, there is a deeply rooted social stigma associated with mental health issues as they view their religion as a source of healing and power, particularly in relation to their mental health [24]. Hence mental illness may be viewed as a consequence of losing faith or deviation from the right path [24]. Muslim women often face discrimination, insensitivity, and health practitioners generally lacked knowledge about their religion and culture, and the specific needs of Muslim perinatal women [25].

### Purpose and significance of the study

Over 35,000 Syrian refugees have recently arrived in Canada, many of them are women [26], and most of childbearing age [14]. Resettlement agencies report a majority of the women they are supporting are pregnant, have a new baby, or a young family [27]. However, little is known about the experiences that Syrian refugee mothers face, their perceptions of mental health, depression in particular, and the challenges they experience after resettlement. Furthermore, previous research suggests that refugee women have high prevalence rates of maternal depressive symptoms, which often coexist with comorbid anxiety symptoms and/or PTSD symptoms [9, 28, 29]. Thus, there is an urgent need to explore refugee women’s experiences of expecting or having a baby after resettlement in Canada from a mental health perspective to inform service providers and policy makers to develop the services and programs that meet the needs of this specific population.

We sought to answer the following questions: 1. How do Syrian refugee women perceive and understand maternal depression? 2. What are the social support needs, challenges, and expectations of perinatal Syrian refugee women? 3. What are the barriers to access maternal mental health services for Syrian refugee women and what determines their help seeking behaviours? 4. How common are maternal depressive symptoms and other comorbid mental symptoms among our sample of Syrian refugee women?

## Methods

### Recruitment and sample

Participants included 12 Syrian refugee women who resettled in Saskatoon, Canada in 2015–2016. Participants were eligible to participate in the study if they were: 1. either pregnant or within 1 year of giving birth, 2. admitted to Canada either as a government-sponsored or a privately-sponsored Syrian refugee, 3. able to speak Arabic or English. Participants were recruited from local outreach programs, which had experienced an influx of Syrian refugee women. We also recruited through local resettlement agencies, maternity wards, religious organizations, and doctor’s offices known to care for refugee families using study posters in both English and Arabic. A list of contact information of all women who expressed interest in our study was collected; however, due to time and financial considerations of this preliminary study, our sample was limited to the 12 participants who appeared first on the list.

### Design

A qualitatively-driven mixed methods research design was used in this study [30]. Purposeful sampling guided our sampling strategy. Ethics approval for the project was obtained from the University of Saskatchewan Behavioural Ethics Review Board and from the Saskatoon Health Region. Informed oral consent was obtained.

### Measures

Written questionnaires included sociodemographic (age, marital status, education, employment, income, housing, primary language in the home, and English fluency), psychosocial (social supports, stressors), as well as obstetrical, and general health factors (gravidity, health status, planned pregnancy, previous pregnancies, their outcomes and complications, medical illnesses and hospitalizations). Questions about the type of delivery and any obstetric complication were included as there is some evidence from the literature that the presence of obstetric complications may be associated with maternal depression [19]. However, due to the small number of women in the postnatal period in our sample, we did not report these findings. Mental health items included history of depression, particularly perinatal depression and treatments received, and screening for depressive symptoms, anxiety symptoms, and PTSD symptoms. Since maternal depression often coexist with anxiety symptoms in general, and with PTSD in refugee populations [9, 28, 29], we sought to screen our participants for these often-comorbid disorders, especially that the EPDS screening tool can also screen for anxiety. We also intended to test the acceptability of the translated version of these screening tools to our participants. Resettlement history before coming to Canada, if in a refugee camp and, if yes, for how long, time since arrival to Canada, accompanying family members, and data about resettlement services received was gathered. All written materials were translated into Arabic.

#### Maternal depressive symptoms

Depressive symptoms were assessed using the Edinburgh Postnatal Depression Scale (EPDS) [31]. The EPDS is one of the most validated screening tools for the detection of perinatal depression that has been translated into many languages [31]. It is a 10-item self-rated measure for a total score of 30 and it has a sensitivity of 0.59–1.00 and specificity of 0.49–1.00 [32], though the sensitivity and specificity may vary across the perinatal period [33, 34]. The validated Arabic version of the EPDS was used to screen the participants for depressive and/or anxiety symptoms with similar levels of internal validity to the English version with a recommended cut off score of 10 or more (α = 0.84) [35–37].

#### Maternal anxiety symptoms

A 3-item anxiety subscale of the EPDS (EDPS-A, items 3, 4, 5) has also been confirmed as a screen for perinatal anxiety symptoms with 4 or more as the recommended cut off score [38, 39].

#### Post-traumatic stress disorder (PTSD)

The Primary Care PTSD screening tool is a four-item screening tool that is a validated PTSD screen for primary care settings with a maximum score of 4 and an optimal cut-off point of 3 or more [40, 41]. It has a sensitivity of 0.78, a specificity of 0.87, a PPV of 0.65, and an NPV of 0.92 [40].

#### Intimate partner violence (IPV)

It has been documented that refugee women may be at higher risk of intimate partner violence, which in turn may increase their vulnerability of developing maternal depression [15, 42]. The short form of Women Abuse Screening tool (WAST) was used [43]. If the participant indicated that she and her partner work out their argument “with great difficulty” and if she described her relationship with her partner as “a lot of tension”, the results indicated a positive screen.

### Focus group

Focus groups (FG) can be used for exploratory and confirmatory inquiry and they have been used successfully in studies with refugee populations [44, 45]. Due to time, cost, and other logistical considerations, all invited study participants showed up and took part in a single FG that lasted approximately 90 min. We conducted the one FG in Arabic in a private room at a Primary Health Centre that the women had been attending for maternal services. Childcare was available by professional caregivers in a room very close by. The discussion was facilitated by the author A. A. who is a mother and an immigrant from Libya who speaks fluent English and Arabic and is a trained physician with cultural competency. A translator who has recently worked with Syrian refugee women in the community was also available during the FG to aid in the translation/interpretation process should there be different dialects or to interpret if a woman needed to leave the FG for any reason. The FG was guided by open-ended questions, which were intended to elicit participants’ perceptions around maternal depression, participants were free to discuss personal experiences if they wanted and each woman was encouraged to elaborate on the most significant answers to her. The author was responsible for keeping the discussion on track and for following up with prompts such as “why…” and “how…” on the important areas of the discussion.

During the FG, participants were invited to discuss the challenges and strategies women take to alleviate stress or solve resettlement issues, the challenges of also being pregnant and/or having a baby. They also discussed the sources and types of support they receive. Participants were also encouraged to discuss their understanding of maternal depression, barriers that may prevent depressed women from seeking help, and factors to help women with maternal depression.

Participants were asked to keep the confidentiality of the other members of the group by not disclosing the contents of discussion outside the group and they were informed that they can withdraw consent for the group discussions at any time. We were aware that the discussion may trigger physical or psychological symptoms, thus we provided them brochures and handouts with contact information of the available resources and local materials related to maternal mental health translated into Arabic. In addition, author A. B. was present and is experienced doing research with perinatal women with mental health problems and the FG was conducted in a primary healthcare center with physicians available onsite. Their findings are reported using pseudonyms not related to their names.

### Data analysis

Thematic analysis was used to analyze data from the FG. Initial coding starts by creating codes, then regrouping codes into categories and grouping categories into sub-themes and themes based on the principles of emergence and inductive analysis [46]. Firstly, the FG data were taped and translated from Arabic to English immediately afterwards by the author A. A. onto another recorder, which was then professionally transcribed. To ensure accuracy of the translated transcripts, transcripts then were checked against audiotapes by A. A. and by the Arabic translator present during the FG. After transcription, A.A. revisited the original audiotapes frequently, whereas all researchers read hard copies of transcripts several times to familiarize themselves with the content of the discussion. Next, the author A.A. generated initial codes and then grouped into and a list of preliminary categories. The list of preliminary categories was then shared with the other authors. Then, regular meetings were held to organize and refine codes and categories into the final sub-themes and themes that will be used to present results of the qualitative data. NVivo software was used for qualitative data management. All three researchers are mothers from diverse cultures and experience, and thus understand the meaning of maternal depression from different perspectives. The author A. A. who recruited participants and facilitated the FG is a mother and an immigrant from the same culture as the study participants. Her fluency in English and Arabic ensured her facilitation of the FG discussion was culturally sensitive and aided her ability to translate and interpret the qualitative data within the context of culture and religion.

Several measures were taken to ensure trustworthiness (rigor) of the analysis [47]. Information about maternal depression and other psychosocial determinants were collected via the focus group and structured questionnaires. Researchers also aimed to provide rich contextual descriptions of the settings, participants, as well as the results of the qualitative analysis. The author A.A. engaged with participants at multiple settings (prenatal as well as postnatal classes) before starting the data collection to build trust and establish rapport with the participants. The researcher used these informal settings as an opportunity to explain the research project, its objectives, the role of researchers, as well as to address any inquiries participants may have, especially as to the potential impact of participation in this research on services they receive. The data collected from the structured questionnaire comprised the quantitative portion of the analysis and they were summarized to provide context for the qualitative analysis and was analyzed the SPSS Statistics 20.0.

## Results

### Sample characteristics

Twelve women were recruited into the study. Two thirds of participants (*n* = 8) were pregnant and a third of them (*n* = 4) were postpartum and their ages ranged from 20 to 37 years (mean = 27.17, SD = 4.9 years). All participants were married, Syrian-born, spoke Arabic as a first language, and had been displaced in a nearby country, i.e., Jordon, Lebanon, or Turkey, before coming to Canada. Five had lived in a refugee camp, but mostly for less than 2 weeks. Most were on social assistance, unemployed before and after coming to Canada (*n* = 10), and had only completed grade 12 (high school diploma) or less (*n* = 9). The majority have been in Canada for less than 8 months (*n* = 11), all were in rented accommodations, and all were living with husband and child/children. Most of them rated their health as fair or good. See Table 1.

**Table 1:** Sociodemographic Characteristics of Participants; [number (proportion)] unless otherwise specified

Characteristics of participants	All participants (Total *n* = 12)	With depressive symptoms (*n* = 7) ^a^	With depressive and anxiety symptoms ^b^ (*n* = 6)	With depressive, anxiety, and PTSD symptoms ^c^ (*n* = 2)
Maternity status				
Pregnant	8 (0.67)	3 (0.43)	3 (0.50)	1 (0.50)
Postpartum	4 (0.33)	4 (0.57)	3 (0.50)	1 (0.50)
Mother’s age (M ± SD)	27.2 ± 4.9 years	24.7 ± 3.4 years	24.8 ± 3.7 years	26.5 ± 5 years
Parity				
2	4 (0.33)	4 (0.57)	4 (0.67)	2 (1.00)
3	1 (0.83)	0 (0.00)	0 (0.00)	0 (0.00)
4	2 (0.17)	2 (0.29)	1 (0.17)	0 (0.00)
5 or more	5 (0.42)	1 (0.14)	1 (0.17)	0 (0.00)
Total number of children in household				
1	3 (0.25)	3 (0.43)	3 (0.50)	1 (0.50)
2	3 (0.25)	1 (0.14)	1 (0.17)	1 (0.50)
4	3 (0.25)	2 (0.29)	1 (0.17)	0 (0.00)
5 or more	3 (0.25)	1 (0.14)	1 (0.17)	0 (0.00)
Time in Canada				
< 6 months	6 (0.50)	3 (0.43)	3 (0.50)	1 (0.50)
7–8 months	5 (0.42)	3 (0.43)	33.3	0 (0.00)
> 8 months	1 0.83)	1 (0.14)	16.7	1 (0.50)
Marital status				
Married	12 (1.00)	7 (1.00)	6 (1.00)	2 (1.00)
Education				
Less than grade 8	5 (0.42)	3 (0.43)	3 (0.50)	1 (0.50)
Between grade 9 and 12	4 (0.33)	1 (0.14)	0 (0.00)	0 (0.00)
Some university/postsecondary education	1 (0.83)	1 (0.14)	1 (0.17)	0 (0.00)
Completed university/postsecondary education	2 (0.17)	2 (0.29)	2 (0.33)	1 (0.50)
Income				
Social assistance	11 (0.92)	6 85.7	5 83.8	1 (0.50)
< 20,000$ annually	1 (0.83)	1 14.3	1 16.7	1 (0.50)
Housing				
Renting	12 (1.00)	7 (1.00)	6 (1.00)	2 (1.00)
Employment before move				
Unemployed	10 (0.83)	6 (0.86)	5 (0.83)	2 (1.00)
Employed	2 (0.17)	1 (0.14)	1 (0.17)	0 (0.00)
Employment Now in Canada				
Unemployed	12 (1.00)	7 (1.00)	6 (1.00)	2 (1.00)
English Confidence				
Not at all confident	2 (0.17)	2 (0.29)	1 (0.17)	0 (0.00)
A little confident	3 (0.25)	1 (0.14)	1 (0.17)	1 (0.50)
Moderately confident	5 (0.42)	3 (0.43)	3 (0.50)	1 (0.50)
Very confident	2 (0.17)	1 (0.14)	1 (0.17)	0 (0.00)
Displaced before ^d^				
Yes	12 (1.00)	7 (1.00)	6 (1.00)	2 (1.00)
Has been in a refugee camp				
Yes	5 (0.42)	4 (0.57)	3 (0.50)	1 (0.50)
No	7 (0.58)	3 (0.43)	3 (0.50)	1 (0.50)
Resettlement services				
Help finding house	12 (1.00)	7 (1.00)	6 (1.00)	2 (1.00)
Language training	9 (0.75)	4 (0.57)	3 (0.50)	1 (0.50)
Help with translation/interpretation	9 (0.75)	4 (0.57)	3 (0.50)	0 (0.00)
Help with health problems	9 (0.75)	5 (0.71)	4 (0.67)	1 (0.50)
Job training	2 (0.17)	1 (0.14)	0 (0.00)	0 (0.00)

Data are percentages unless otherwise indicated

^a^total EPDS score of 10 or greater

^b^total EPDS score of 10 or greater and EPDS anxiety subscale score of 4 or greater

^c^total EPDS score 10 or greater, EPDS anxiety subscale score of 4 or greater, and primary care PTSD scale score of 3 or greater

^d^All has been displaced to a country nearby Syria (Jordon, Lebanon, Turkey)

### Mental health

More than half (7/12) of participants screened positive for possible depression (EPDS score 10 or greater) and half of them (6/12) screened positive for possible anxiety (EPDS anxiety subscale score of four or greater). Their total EPDS scores ranged from 0 to 19 (mean = 8.75.0, SD = 5.85) and their EPDS anxiety subscale scores ranged from 0 to 8 (mean = 3.66.7, SD = 2.5). Those with both depression and anxiety symptoms were young (3/6 were <25 years), half of them of them were pregnant and half were postpartum, three of them had history of depression during a previous pregnancy/postpartum, and two of them screened positive for PTSD.

Four women had a previous history of depression and three of those indicated they had a history of antenatal and postpartum depression. Most indicated that they felt stressed, mainly related to the upcoming birth of the baby for most of those who were pregnant. All of the women indicated that they had someone in their lives for support, mostly their partner, and all of them responded to the question about intimate partner violence negatively.

### Qualitative findings

Three themes emerged: 1) Understanding of maternal depression; 2) Protective factors for mental health; and 3) Barriers to mental health services.

#### Understanding of maternal depression

Despite the high proportion of women with high EPDS scores, participants did not disclose any depressive symptoms during the FG, and used other terminologies instead, such as being bored or tired, to describe their feelings. Moreover, participants had many misconceptions around maternal depression; its meaning, its causes, and how common it is in Syrian women.

When asked if they knew what is maternal depression, most of the women had heard of it. They tended to describe extreme cases as “depression” whereas they used terms such as bored or tired, to label milder depressive symptoms.

Marwa: *“Sometimes you feel bored. It’s not depression, it’s just that I might be bored and my mood will change and this is something normal. But depression means being sick and requiring a treatment. I’ve never had that, but I have heard of it. I’ve heard that many women will experience this either before birth or postpartum…*. *The woman will be sick to the point where she can’t see anyone or speak to anyone and will need a physician.*”

The women ascribed maternal depression to a range of causes including having a girl baby whilst expecting a boy, giving birth away from family, baby’s death, or worries about the baby’s birth, especially in a first pregnancy.

Afnan*: “If you ask any Syrian person why a woman is depressed, they would say “being away from her family, alienation”.”*

Yusra*: “I had a depression before I was pregnant in Syria and I was injured. ...After birth, I wasn’t able to walk because of the injury, so I became depressed. … I was very depressed but I had a hope because I wanted to feel better for my girl. After 3 months, she died, and I was very depressed.”*

Most of the participants said that maternal depression happens less in the Syrian women, compared to other women. They believed that Syrians in general love to have children and hence everybody is very happy and pleased after birth. One woman described the strong support that was provided to women in Syria around birth as a possible factor that may explain why she thought depression is less in Syrian women.

Eman: “*It’s less. Everybody is happy. I think that it is less. Whenever I hear about a woman gives birth, she will be happy and will be relieved. It is for sure less. ... the women will be with her family and will be happy. She will have a baby. Things were very good in Syria before, so maybe because of that.”*

#### Protective factors for mental health

Despite all their difficulties, the women described strategies that protected their mental health. The women defined support as the social support they receive around the time of birth, either during labor or afterward, with emphasis on emotional support.

Joud*: “You feel that you aren’t alone and you will feel that someone is standing beside you.…Like when you are going to give birth, you really need your mother, your aunt or somebody.”*

However, they also appreciated other forms of support.

Kadiga*: “There is also the support for the other children…. Also the help with housework and also for the new baby, who will need care. The mother will be depressed/concerned about whether to take care of the baby, her health, other children, or for the house.”*

Reem*: “I used to have my mother and my sisters around me during labor, but I will be alone here. This is a big thing for me. I am displaced and all my family is away and this is something so big for me.”*

When they were asked about things that might help a woman with depression, all thought that reconnecting them with their family would be most beneficial.

Reem*: “It should be like we can go and visit them or the family can come and visit us. This way we would not feel like a bird in a cage or imprisoned…. This is the most important and critical issue here. This is what can cause depression.”*

Participants greatly appreciated the support from Canada and welcoming them by providing them with housing, medical care, and financial support.

Afnan*: “Feeling of hope, feeling that there is a good future here…. Hope and feeling safe.”*

Spiritual practices, such as prayer and reading the Holy Quran were mentioned as a source of support and strength for all the women.

Joud*: “For us, when somebody has a low mood, they will open the Quran and read.”*

Participants believed that support programs would be useful for their mental health, but suggested that to avoid stigma from their community programs should be named in a way that does not refer directly to depression but rather wellness.

Afnan*: “Programs for Syrian women to speak about their situation and about everything related to them, like the challenges they face.... Make a program for them under another name, like for example “towards a better life”, so the topic of discussion will be depression, but in front of the general public, it’s not, so they can talk about depression and they will not be shy to participate, and they can talk freely.”*

Participants felt that exercises like walking and swimming would be helpful for their mental health. However, they felt that all recreational programs in Canada are mixed “both gender”, which they thought hindered their engagement in such activities. As Muslim women who are covering their hair and body, are used to have special places for women where they can participate easily in various outdoor and indoor activities.

Reem*: “We have to make sure that we have these kinds of things are not mixed-like men and women. There should be something special for the women.... So, men have a lot of things that are set for them, but there are no special programs for the women with hijab.… So here, I miss swimming and I miss special farms/gardens for us. This is something that I really miss here in Canada.”*

Meditation activities such as yoga, that are very popular in western culture for depression [48] were not well-known in the Syrian culture, and most of the participants have never engaged in such activities.

Participants were strongly against the use of medication for depression, with only a few saying that medication may be needed in severe cases, but then only as the last treatment choice. Some however, believed that medication may help a woman to get enough sleep.

#### Barriers to mental health services

Participants described two major obstacles that may prevent Syrian refugee women from seeking or accessing mental health services; stigma of mental health and privacy concerns.

Marwa: *“I might have depression… If I went to a psychiatrist, they would say that she “went crazy”. So, society would think that she is a psychiatric patient and is getting treatment. …. She might only have a low mood and want someone to talk to, someone to make her aware, but no, they will think immediately that she is crazy.”*

Even though there is that stigma associated with seeking mental health services, some felt strong enough to seek help, if they needed to.

Kadiga*: “If I have depression, I will not have a problem going to a psychiatrist and seeking help. I’m sure that they will speak about me and say that I’m crazy but this doesn’t matter to me. What is important to me, is to treat myself so I can live comfortably with my family.”*

Participants also spoke about the possibility that a husband may prevent the woman from seeking help or from disclosing her symptoms, even to her family, for fear of social stigma, although they denied that this had happened to them.

Eman*: “For every woman, her husband’s way of thinking is different…. Some men, they don’t like that their wives to speak, even to her neighbors. It’s a form of privacy.”*

Concerns about privacy, confidentiality, and whether other people, especially Syrians, would know that a woman is seeking help for her mental health were also cited by participants as possible barriers. Nonetheless, they felt that privacy concerns were less of a concern here in Canada than it was in Syria or in other Middle Eastern countries.

*Yusra: “Maybe she will be shy that somebody will know, somebody will know her name, her story, her life, how she is living, etc. She would think that this is private life.… Her privacy might prevent her from this…. She might think that this is her private life.”*

Language difficulties also impacted their privacy, especially when seeking healthcare services. Women said that they would be willing to share their symptoms with a healthcare provider, but not necessarily in the presence of an interpreter, especially where there is a small community of Arabic speaking people.

Yusra*: “If you speak with a psychiatrist, you would speak normally, but if there is an interpreter as a mediator, and this person might speak about what you said, and now like you have told your story to this and may be this mediator will tell everybody in Canada.”*

## Discussion

This preliminary study demonstrates that maternal depression is an important feature in Syrian refugee women who have recently resettled in Canada. Almost two thirds of participants screened positive for possible depression, which is close to what was previously reported (rates as high as 57% in developing countries) [2]. It is evident that perinatal Syrian refugee women are exposed to many factors that can impact mental health such as lack of social support, language and economic factors [3, 6, 16, 17]; and that experiencing all of these factors combined, along with experiences that may be unique to refugee women, likely increases their vulnerability to maternal depression.

The experience of migration and resettlement may influence Syrian refugee women’s experiences of pregnancy and motherhood jeopardizing their overall mental well-being. The migration process may have added a huge amount of stress and uncertainty to those women’s lives, raising their vulnerability to depression, especially around the time of giving birth. The refugees’ experience of migration has its unique aspects, as most Syrian refugees resettled in Western countries have been displaced, either inside Syria or in one of the nearby countries, before resettlement. Most of our study participants indicated similar pre-migration experiences before resettling in Canada, a fact that has been documented in the literature as a risk factor for mental illness [49]. A Canadian study of refugees resettled in a mid-sized Canadian city found that multiple forced migrations increases the risk of emotional distress and affects social and cultural adaptation [49].

Among refugee populations, the literature has documented a prevalence rate of PTSD as high as 10% as described in a systematic review that included 7000 refugees resettled in Western countries [28]. Only two participants (12%) in the current study screened positive for PTSD symptoms, which is comparable to these results. However, it is possible that women with PTSD symptoms may be traumatized to the point that they avoid triggers of their past traumatic experiences, and thus were less likely to participate in research studies.

Despite the high proportion of women with depressive symptoms reflected in their EPDS scores, participants did not disclose any depressive symptoms during the FG, and used other terminologies instead, such as being bored or tired, to describe their feelings. This could be linked to how negatively Syrian people perceive mental illness [50]. Syrian refugee women make a close connection between being happy postpartum and loving the baby, which may make it difficult for them to share their feelings of depression, especially within a group of their peers. Four participants acknowledged having a history of depression, but it was difficult for them to admit this within the group until later in the discussion, and explained major life events, such as injury or death of the baby, as causing their depression. Our results are in keeping with previous research which showed that women from a variety of cultural backgrounds are against the use of medication for depression [51, 52].

Consistent with other research, the women emphasized stigma and privacy concerns as the strongest constraints to their accessing to mental health care [6, 20, 22]. As documented by O’Mahony, cultural beliefs can have both positive and negative impact on refugee women’s mental health [6]. The stigma and labelling of mental illnesses within the Syrian community was presented in this study as one of the major determinant of whether to seek help or struggle alone for the Syrian refugee women.

While the literature indicates that relationship difficulties contribute to the high vulnerability for maternal depression in refugee women [15], our participants did not specify relationship problems as an issue for them during their discussion or on their responses to the abuse questions. This may be because of the difficulty of discussing it within a group, but it may also be because these women indicated that their husband was their main supporter and they could count on him no matter what. When family support and separation was raised in the FG, some women started crying and left the room briefly, which showed how sensitive and significant family support is for them, as it has been found to be in other refugee women [6, 17]. With all of the difficulties that the women may be experiencing, the birth of the baby was their major source of stress for the pregnant ones, which shows how difficult it is for them to give birth in Canada.

Although this was not the first birth for all the women, it was the first time giving birth away from their families and they talked extensively how their families, particularly female relatives, mostly their mother, would provide them with support around birth if still in Syria, which may raise the question of whether the husband is prepared for the new role of being the main supporter of the wife around birth, as they have not been used to such a responsibility. Reports also highlight such a strong relationship between Arabic women with their mother, and the impact of the disruption of such a relationship on their mental health and well-being [17].

### Limitations

As this was a preliminary study, our sample was rather a small sample of Syrian refugee women located in one city in Canada, and the data were collected from a single FG. Thus, readers should exercise caution when generalizing our findings to other Syrian refugee women or other refugee populations. While all efforts were made to ensure accuracy of the translation of data from Arabic to English, through Arabic speaking researcher and translator and a validated tool, the exact meaning of specific words may have different meaning when translated into a different language. The qualitative data collection involved discussing such sensitive issues within a group setting and in the presence of a translator may have also limited our findings.

### Implications

Our findings illustrate the need for raising awareness among Syrian refugee women, their families, and their communities, about the symptoms, causes, treatment, and consequences of maternal depression. This can be done in prenatal and postnatal classes, language training centers, resettlement agencies, community centers, religious organizations, public health clinics, and doctor’s offices through presentations, group discussions, and through displaying materials in Arabic languages about maternal depression, but the women and their families need to be aware and to connect with the available services. Screening refugee women for mental health problems, providing referral, and offering transportations and childcare are also essential elements to improve access to health and social services.

Support programs and gendered recreational group classes may help women to form social connections and build a social network to make them feel supported in their new communities, besides the positive impact exercise has on mental health. Family reunification was also seen as an essential element to their support and hence connecting refugee women with their families, especially mothers, during the time of childbirth might be necessary for their mental well-being. Trained interpreters who have clear guidelines regarding client’s privacy and confidentiality are essential as well as bilingual health care providers, but also support to improve the women’s language skills to promote their integration in the culture and comfort with health care services.

Clinicians should also recognize how the social determinants of health among refugee women, such as social isolation, poverty, education, and economic status, besides past traumatizing experiences, may affect their mental well-being and may make refugee women more susceptible to maternal depression. Syrian refugee women may mask their depression symptoms by smiling or admitting to being tired rather than expressing depression symptoms as other women do; therefore, universal depression screening using the validated Arabic version of EPDS would be valuable. In addition, healthcare providers need to be watchful for other terminologies the Syrian women may use to express their mental health symptoms and the community supports available to them. Mental health treatment options should be discussed in a sensitive and culturally appropriate way to ensure cooperation.

Larger mixed methods studies are needed to inform program and policy development about the magnitude of the problem of maternal depression and other related mental health problems among Syrian refugee women, their social and health needs around the time of birth, and the barriers these women face when accessing mental health services.

## Conclusion

Maternal depression is an important feature in Syrian refugee women recently resettled in Canada. Education of Syrian women is needed about symptoms, effects, causes, and treatment, of mental health disorders. Reuniting these women with their families and engaging them in culturally appropriate support programs may improve their mental health outcomes. Care providers need to realize that because a refugee woman is smiling, does not mean she may not be experiencing depressive symptoms and that culturally sensitive screening and assessment may be necessary to determine women who need further investigation and treatment supports. Larger studies are needed to inform program and policy development.

## Additional files

Additional file 1: Structured questionnaire. English language versions of the structured questionnaires developed specifically for use in this study. It included the Primary Care Post-traumatic stress disorder (PTSD) screening tool, the short form of Women Abuse Screening tool (WAST), the Edinburgh Postnatal Depression Scale (EPDS), sociodemographic questions, as well as questions about past psychiatric history, sources of stress, and sources of support. (DOCX 42 kb)

Additional file 2: Focus group topic guide. Focus group discussion guides developed specifically for use in this study. (DOCX 18 kb)

## Abbreviations

EPDS: Edinburg Postnatal Depression Scale
EPDS-A: Edinburg Postnatal Depression Scale-Anxiety subscale
FG: Focus Groups
IPV: Intimate Partner Violence
PTSD: Post-Traumatic Stress Disorder
WAST: Women Abuse Screening Tool
