# Supplementary material for: Maternal depression in Syrian refugee women recently moved to Canada: a preliminary study
Source: BMC Pregnancy Childbirth. 2017 Jul 24;17:240. doi: 10.1186/s12884-017-1433-2 (PMC5525250; doi:10.1186/s12884-017-1433-2)
Supplement: Supplementary file 1 — Structured questionnaire. English language versions of the structured questionnaires developed specifically for use in this study. It included the Primary Care Post-traumatic stress disorder (PTSD) screening tool, the short form of Women Abuse Screening tool (WAST), the Edinburgh Postnatal Depression Scale (EPDS), sociodemographic questions, as well as questions about past psychiatric history, sources of stress, and sources of support. (DOCX 42 kb) [file 12884_2017_1433_MOESM1_ESM.docx]

**Structured Questionnaire**

**Thank you for agreeing to answer these questions.**

1. **Please underline the answer, which comes closest to how you have felt in the past 7 days ^a^**

I have been able to laugh and see the funny side of the things:

- As much as I always could
- Not quite so much now
- Definitely not so much now
- Not at all

I have looked forward with enjoyment to things:

- As much as I ever did
- Rather less than I used to
- Definitely less than I used to
- Hardly at all

I have blamed myself unnecessarily when things went wrong:

- Yes, most of the time
- Yes, some of the time
- Not very often
- No, never

I have been very anxious or worried for no good reason:

- No, not at all
- Hardly ever
- Yes, sometimes
- Yes, very often

I have felt scared or panicky for no very good reason:

- Yes, quite a lot
- Yes, sometimes
- No, not much
- No not at all

Things have been getting on top of me:

- Yes, most of the time I have not been able to cope at all
- Yes, sometimes I haven’t been coping as well as usual
- No, most of the time I have coped quite well
- No, I have been coping as well as ever

I have been unhappy that I have had difficulty sleeping:

- Yes, most of the time
- Yes, sometimes
- Not very often
- No, not at all

I have felt sad or miserable

- Yes, most of the time
- Yes, quite often
- Not very often
- No, not at all

I have been so unhappy that I have been crying:

- Yes, most of the time
- Yes, quite often
- Only occasionally
- No, never

The thought if harming myself has occurred to me:

- Yes, quite often
- Sometimes
- Hardly ever
- Never

1. **In your life, have you ever had any experience that was so frightening, horrible, or upsetting that, in the past month, you: ^b^**
2. Have had nightmares about it or thought about it when you did not want to? ❑ **Yes** ❑ **No**
3. Tried hard not to think about it or went out of your way to avoid situations that reminded you of it? ❑ **Yes** ❑ **No**
4. Were constantly on guard, watchful, or easily startled? ❑ **Yes** ❑ **No**
5. Felt numb or detached from others, activities, or your surroundings? ❑ **Yes** ❑ **No**
6. **Do you have someone to turn to for support? ^c^** ❑ **Yes** ❑ **No If yes,** who gives you support?

 Partner/husband ❑ Mother ❑ Friend ❑ Female relatives ❑ Other, who? ____________

1. Which of these people gives you the most support? ____________
2. Can you count on that person to care about you no matter what? ❑ **Yes** ❑ **No**
3. **Do your moods go up and down? ^c^**  ❑not at all ❑ several days ❑ more than ½ the days ❑nearly every day
4. **Do you have mood swings that occur for no reason? ^c^** ❑not at all ❑ several days ❑ more than ½ the days ❑nearly every day
5. **Do you have a history of depression? ^c^ ❑Yes ❑No**  when**_____**

Were you treated? ❑Yes❑No With medication? ❑Yes ❑No

1. **Did you have depression in a previous pregnancy? ^c^** ❑ **Yes** ❑ **No** when**____**

Were you treated? ❑Yes❑No With medication? ❑Yes ❑No

1. **Have you had postpartum depression? ^c^ ❑Yes ❑No** when_**___**

Were you treated? ❑Yes❑No With medication? ❑Yes ❑No

1. **What things are causing you the most stress right now? ^c^**

❑ nothing right now

**if yes, what**

❑ partner/relationship ❑ not enough money ❑ children ❑ family

❑ where I live ❑ health of my baby ❑ birth of my baby ❑ my health

❑ work ❑ school ❑Other_______

1. **In general, how would you describe your relationship: ^d^** ❑ a lot of tension ❑ some tension ❑ no tension?
2. **Do you and your partner work out arguments ^d^** ❑ with great difficulty ❑ some difficulty ❑ no difficulty?

**Questions about you ^c^**

1. **What is your date of birth (dd/mm/yyyy): ______/_______/__________**
2. **What is your marital status?**

- Single
- Married/ Partnered relationship
- Widowed
- Divorced/separated

1. **What is the highest grade that you have completed?**

- Grade 8 or less
- Grade 9-11
- Completed grade 12
- Some postsecondary or university education
- University/postsecondary graduate

1. **What is your country of birth: _____________________________________**
2. **Have you been displaced before coming to Canada? If yes please specify**

**_____________________________________**

1. **Have you ever lived in a refugee camp? If yes, for how long? ________where? _____**
2. **How long have you been in Canada? _____________________________________**
3. **Who did you come to Canada with? _____________ _______________________**
4. **Have you received any of the following resettlement services since you arrived in Canada? Please check all that apply**

- language training
- job training
- help with translation
- help finding house
- help with health problems
- help with legal issues

1. **Which language do you speak at home? ______________________________________________**
2. **How confident are you using English?**

- not at all
- a little
- moderately confident
- very confident

1. **What is your employment history before coming to Canada (choose one)**

- Unemployed
- Casual worker
- Part time
- Full time
- Other _______________

1. **What is your employment status at the moment (choose one)**

- Unemployed
- Casual worker
- Part time
- Full time
- Other ________________

1. **Which of the following best describes your current living situation?**

- Homeowner
- Renter
- Living with others, but not paying rent or mortgage
- Living with others and assisting in paying rent or mortgage

1. **How many adults are living in the home? _______________________**
2. **How many children are living in the home? _______________________**
3. **What is your total annual (per year) family income (choose one)**

- Social assistance
- less than $20,000
- $20,000 - $40,000
- >$40,000

1. **How would you rate your overall health today?**

- Excellent
- Very good
- Good
- Fair
- Poor

1. **How many pregnancies have you had?** ❑ **1** ❑ **2** ❑ **3** ❑ **4** ❑ **5** or more
2. **How many children are living including the present baby (if the baby was born)? __________**
3. **Did you plan this pregnancy?** ❑ **Yes** ❑ **No** ❑ **Sort of**
4. **If your baby is born, where was it born?______________**
5. **Did you have any pregnancy or labour complications^e^:** ❑ **Yes** ❑ **No**

If yes, please explain? _____________________________________________________________________________________

1. **What was the type of delivery?^e^** (Please choose one)

❑ Spontaneous vaginal ❑ Assisted vaginal (vacuum or forceps) ❑ Caesarean Section

____________________________________________________________________________________

1. **Child’s health information**

Date of birth of your last baby: ___________ Sex of baby: ❑ Male ❑ Female Birth weight: _______

1. **Did the baby have any complications at birth?^e^** ❑ **Yes** ❑ **No**

If yes, please explain _____________________________________________________________________________________

1. **Is the baby being breastfed?** ❑ **Yes** ❑ **No**  ❑ **breast and formula**
2. **What is the overall health status of the baby now?**

❑ Excellent ❑ Very good ❑ Good ❑ Fair ❑ Poor

**Thank you for your time. Is there anything else you would like to tell us about how you feel, your overall health or your pregnancy or birth experience?**

_____________________________________________________________________________________

**^a^** The Edinburgh Postnatal Depression Scale (EPDS) is one of the most validated screening tools for the detection of perinatal depression that has been translated into many languages [1]. It is a 10-item self-rated measure for a total score of 30 and it has a sensitivity of 59–100% and specificity of 49–100% [2]. The validated Arabic version of the EPDS was used to screen the participants for depressive and/or anxiety symptoms with similar levels of internal validity to the English version with a recommended cut off score of 10 (α = 0.84) [3-5]. A 3-item anxiety subscale of the EPDS (EDPS-A, items 3, 4, 5) has also been confirmed as a screen for perinatal anxiety symptoms with 4 or more as the recommended cut off score [6, 7].

**^b^** The Primary Care Post-traumatic stress disorder (PTSD) screening tool is a four-item screening tool that is a validated PTSD screen for primary care settings with an optimal cut-off point of three [8, 9]. It has a sensitivity of 0.78, a specificity of 0.87, a PPV of 0.65, and an NPV of 0.92 [8].

**^c^** Sociodemographic questions, as well as questions about sources of stress and support have been utilized within our research group through various studies [6, 10-13].

**^d^** Previous research suggests that refugee women are at high risk of relationship problems and intimate partner violence (IPV) [14]; thus, we screened our participants for IPV. The short form of Women Abuse Screening tool (WAST) is a validated screening tool for IPV [15]. If the participant indicated that she and her partner work out their argument “with great difficulty” and if she described her relationship with her partner as “a lot of tension”, the results indicated a positive screen.

^e^ Questions about the type of delivery and any obstetric complication were included as there is some evidence from the literature that the presence of obstetric complications may be associated with maternal depression [16]. However, due to the small number of women in the postnatal period in our sample, we did not report these findings.

**References:**

1. Cox JL, Holden JM, Sagovsky R: **Detection of postnatal depression. Development of the 10-item Edinburgh Postnatal Depression Scale**. *British Journal of Psychiatry* 1987, **150**:782-786.

2. Obstetricians TACo, Gynecologists: **Screening for perinatal depression: Committee Opinion No. 630**. *Obstetrics and Gynecology* 2015, **125**(630):1268-1271.

3. Ghubash R, Abou-Saleh MT, Daradkeh TK: **The validity of the Arabic Edinburgh Postnatal Depression Scale**. *Social Psychiatry and Psychiatry Epidemiology* 1997, **32**:474-476.

4. Matthey S, Barnett B: **Translation and validation of the Edinburgh Postnatal Depression Scale into Vietnamese and Arabic**. *Perspectives on transcultural mental health* 1997:77-84.

5. Barnett B, Matthey S, Gyaneshwar R: **Screening for postnatal depression in women of non-English speaking background**. *Archives of women's Mental Health* 1999, **2**(2):67-74.

6. Bowen A, Bowen R, Maslany G, Muhajarine N: **Anxiety in a socially high-risk sample of pregnant women in Canada**. *Canadian Journal of Psychiatry* 2008, **53**(7):435-440.

7. Matthey S, Fisher J, Rowe H: **Using the Edinburgh postnatal depression scale to screen for anxiety disorders: Conceptual and methodological considerations**. *Journal of affective disorders* 2013, **146**(2):224-230.

8. Prins A, Ouimette P, Kimerling R, Camerond RP, Hugelshofer DS, Shaw-Hegwer J, Thrailkill A, Gusman FD, Sheikh JI: **The primary care PTSD screen (PTSD): development and operating characteristics**. *Primary Care Psychiatry* 2004, **9**(1):9-14.

9. Freedy JR, Steenkamp MM, Magruder KM, Yeager DE, Zoller JS, Hueston WJ, Carek PJ: **Post-traumatic stress disorder screening test performance in civilian primary care**. *Family practice* 2010, **27**(6):615-624.

10. Bowen AN, Bowen RC, Muhajarine N: **Mood variability in pregnant and early postpartum women**. *Journal of Obstetrics and Gynecology of Canada* 2012.

11. Bowen A, Muhajarine N: **Prevalence of antenatal depression in women enrolled in an antenatal outreach program in Canada**. *Journal of Obstetric, Gynecologic, and Neonatal Nursing* 2006, **35**(4):491-498.

12. Bowen A, Stewart N, Baetz M, Muhajarine N: **Antenatal depression in socially high-risk women in Canada**. *Journal of Epidemiology and Community Health* 2009, **63**:414-416.

13. Bowen A, Rahman K: **Examining the Factors that Moderate and Mediate the Effects on Depression during Pregnancy and Postpartum**. *Journal of Pregnancy and Child Health* 2014, **01**(2):1-8.

14. O'Mahony J, Donnelly T: **How does gender influence immigrant and refugee women's postpartum depression help‐seeking experiences?** *Journal of psychiatric and mental health nursing* 2013, **20**(8):714-725.

15. Rabin RF, Jennings JM, Campbell JC, Bair-Merritt MH: **Intimate partner violence screening tools: a systematic review**. *American journal of preventive medicine* 2009, **36**(5):439-445. e434.

16. O'Hara MW, Swain AM: **Rates and risk of postpartum depression - a meta-analysis**. *International Review of Psychiatry* 1996, **8**:37-54.
