# Supplementary material for: Maternal depression in Syrian refugee women recently moved to Canada: a preliminary study
Source: BMC Pregnancy Childbirth. 2017 Jul 24;17:240. doi: 10.1186/s12884-017-1433-2 (PMC5525250; doi:10.1186/s12884-017-1433-2)
Supplement: Supplementary file 2 — Focus group topic guide. Focus group discussion guides developed specifically for use in this study. (DOCX 18 kb) [file 12884_2017_1433_MOESM2_ESM.docx]

**Focus group topic guide**

- Greetings and brief description of the study.
- The researcher will acknowledge the complexity of the Syrian experience, in terms of conflict and displacement, interrupting the traditional/cultural experiences, and the likelihood that many people may have spent time in other countries (adjacent to Syria) before coming to Canada.
- The purpose of this focus group discussion is to help us understand how Syrian refugee women perceive and understand maternal depression and the important health needs, social support needs, challenges, and the expectations of Syrian refugee women during pregnancy and postpartum after their resettlement in Canada.
- The researcher will remind women that all things discussed in the focus group and the membership of the group is confidential.

1. Overall, how do you feel about your experience of being pregnant/having a baby in Saskatoon?
2. Do you feel you are/were adequately prepared to take care of your baby on your own?
3. Who are the people who give you support in your life?
   1. Where are they?
4. After the baby, what are/were the major things for which you need support?
5. Have you heard of depression in pregnant/postpartum women?
   1. Do you think it happens more or less often in women like yourself? Why or why not?
6. Have you received any information about feelings during pregnancy/maternal depression or postnatal depression since being in Saskatoon?
   1. What was this information?
   2. Did you find it helpful to you?
   3. Would you like more information about this?
7. What things do you think would be helpful for refugee women with maternal depression? *(probes following exhaustion of discussion from women: who, what, where, when, why: e.g. Praying, husband, friends, yoga, medications, written information, a support group, walking, etc.)*
   1. Do these resources exist in Saskatoon?
   2. If yes, are they accessible for the Syrian refugee women? Have you accessed them?
8. What factors might prevent women with maternal depression from seeking help from health professionals like counsellors? (*probes following exhaustion of discussion from women:: stigma, language, time, doesn’t know they exist, does not understand the role of counsellor, transport, socially unacceptable to go outside group, husband may feel uncomfortable, etc.)*
9. What could the community & health providers do to better support refugee women with maternal depression and their families?
10. Would you like to talk about anything else that is important to you or you feel we haven’t talked about related to mother’s feelings and support services?

Thank you
